# Microbial Methane Production Associated with Carbon Steel Corrosion in a Nigerian Oil Field

**DOI:** 10.3389/fmicb.2015.01538

**Published:** 2016-01-11

**Authors:** Jaspreet Mand, Hyung S. Park, Chuma Okoro, Bart P. Lomans, Seun Smith, Leo Chiejina, Gerrit Voordouw

**Affiliations:** ^1^Petroleum Microbiology Research Group, Department of Biological Sciences, University of CalgaryCalgary, AB, Canada; ^2^Cormetrics Ltd.Calgary, AB, Canada; ^3^Department of Biology, Microbiology and Biotechnology, Federal University, Ndufu-Alike, IkwoEbonyi, Nigeria; ^4^Shell Global Solutions InternationalRijswijk, Netherlands; ^5^Shell Nigeria Exploration and Petroleum CompanyLagos, Nigeria; ^6^Shell Petroleum Development Company of NigeriaPort Harcourt, Nigeria

**Keywords:** microbially influenced corrosion, microbial community, pyrosequencing, methanogen, acetogen, biofilms

## Abstract

Microbially influenced corrosion (MIC) in oil field pipeline systems can be attributed to many different types of hydrogenotrophic microorganisms including sulfate reducers, methanogens and acetogens. Samples from a low temperature oil reservoir in Nigeria were analyzed using DNA pyrotag sequencing. The microbial community compositions of these samples revealed an abundance of anaerobic methanogenic archaea. Activity of methanogens was demonstrated by incubating samples anaerobically in a basal salts medium, in the presence of carbon steel and carbon dioxide. Methane formation was measured in all enrichments and correlated with metal weight loss. Methanogens were prominently represented in pipeline solids samples, scraped from the inside of a pipeline, comprising over 85% of all pyrosequencing reads. Methane production was only witnessed when carbon steel beads were added to these pipeline solids samples, indicating that no methane was formed as a result of degradation of the oil organics present in these samples. These results were compared to those obtained for samples taken from a low temperature oil field in Canada, which had been incubated with oil, either in the presence or in the absence of carbon steel. Again, methanogens present in these samples catalyzed methane production only when carbon steel was present. Moreover, acetate production was also found in these enrichments only in the presence of carbon steel. From these studies it appears that carbon steel, not oil organics, was the predominant electron donor for acetate production and methane formation in these low temperature oil fields, indicating that the methanogens and acetogens found may contribute significantly to MIC.

## Introduction

Oil production is one of the most important factors contributing to the economic growth of Nigeria (Ogwumike and Ogunleye, 2008). According to the Organization of the Petroleum Exporting Countries (OPEC), the oil and gas sector accounts for close to 35% of the gross domestic product of Nigeria (OPEC Annual Statistical Bulletin, 2015^1^). The majority of the development of this resource occurs in the Niger delta area in southwestern Nigeria.

Microbial activity in oil reservoirs is common and can impact oil and gas production. In fact, some problems associated with hydrocarbon recovery can be attributed to microbial activity (Voordouw, 2011). In particular, microorganisms may be responsible for different mechanisms that lead to pipeline corrosion failures. Pipeline corrosion can have serious economical implications including production shutdown and the need for infrastructure replacement (Jones, 1996). Corrosion linked to the activity of microorganisms is termed microbially influenced corrosion (MIC) and is often caused by hydrogenotrophic microorganisms. These include the sulfate-reducing bacteria (SRB), which are able to reduce sulfate to sulfide. It has long been proposed that these bacteria use the hydrogen formed on the surface of pipeline metal to reduce sulfate (Von Wolzogen Kühr and Van der Vlugt, 1934). More recently, SRB that are able to directly use iron as electron donor for sulfate reduction have been discovered (Enning et al., 2012). Furthermore, SRB cause increased sulfide levels in petroleum product, known as souring (Gieg et al., 2011). The microbially produced sulfide can precipitate with iron as iron sulfides; semiconductive minerals that play a role in steel corrosion (Enning et al., 2012). In addition to SRB, methanogenic archaea and acetogenic bacteria have also been reported to be capable of steel corrosion. Hydrogenotrophic methanogens and acetogens are able to use hydrogen on the surface of steel pipelines to reduce bicarbonate to either methane or acetate, respectively (Daniels et al., 1987; Mori et al., 2010; Mand et al., 2014). Recently, strains capable of using the steel directly as an electron donor have also been isolated (Dinh et al., 2004; Uchiyama et al., 2010; Kato et al., 2014; Siegert et al., 2015). Consortia involving these microorganisms have been implicated in MIC in numerous environments (Zhang et al., 2003; Davidova et al., 2012; Usher et al., 2014, 2015).

The production facility we studied was the Obigbo oil field, a site recently used for MIC studies (Okoro et al., 2014). The Obigbo reservoirs are injected with low-sulfate groundwater for pressure support to stimulate oil production. The production fluids from this site are then transported to the Bonny Oil and Gas Terminal (BOGT) for export. The Obigbo reservoir temperature is close to 40°C and concentrations of sulfate in formation water have historically been low (0–7 ppm) (Okoro et al., 2014). As a consequence of the low availability of sulfate from both the injection as well as the formation water, neither souring, nor MIC caused by SRB is considered a severe threat. Hence this field does not employ nitrate injection as a means to control SRB activity. Instead, previous research has demonstrated that methanogenic archaea are not only present, but active within the Obigbo reservoir and production facilities (Okoro et al., 2014). The role these microorganisms are playing remained unspecified, since in addition to the growth using carbon steel, these methanogens may also be able to produce methane through the syntrophic breakdown of oil organics within the field (Widdel and Rabus, 2001; Gieg et al., 2014). Syntrophy involves the breakdown of hydrocarbons by fermentative microorganisms to form compounds such as formate, acetate or hydrogen (Sieber et al., 2012). Subsequently, these compounds are used by methanogenic archaea to produce methane (Sieber et al., 2012; Gieg et al., 2014). The interactions between different groups of microorganisms ensures that the concentration of small intermediary compounds remains low, and therefore syntrophy is a mutually beneficial manner of growth (Morris et al., 2013).

As part of regular maintenance at this field, pipelines are mechanically cleaned using brushes and pigs to rid the carbon steel surfaces of sludge, scale and deposits that may have formed (Videla, 2002). These scrapings, or pipeline pigging samples, consist of formation sands, organic hydrocarbons, inorganic minerals, corrosion products such as iron sulfides and also microbial biofilms. This resulting scale/wax/debris can therefore be a valuable sample; often overlooked in favor of easy to obtain water samples, to determine corrosion products, corrosion risks and corrosion mechanisms by microorganisms (Wrangham and Summer, 2013). A previous microbial study of the Obigbo field indicated that biological activity exists in such deposits and it is important to understand how these solids scraped from pipeline walls, may be enhancing MIC (Okoro et al., 2014).

In our study, we obtained both water samples and pipeline pigging solids samples from the Obigbo field. The objective was to determine the potential for biocorrosion in these samples and further understand how hydrogenotrophic acetogenic and methanogenic microorganisms may be playing a role in MIC.

## Materials and methods

### Sampling procedure

**Figure 1:**
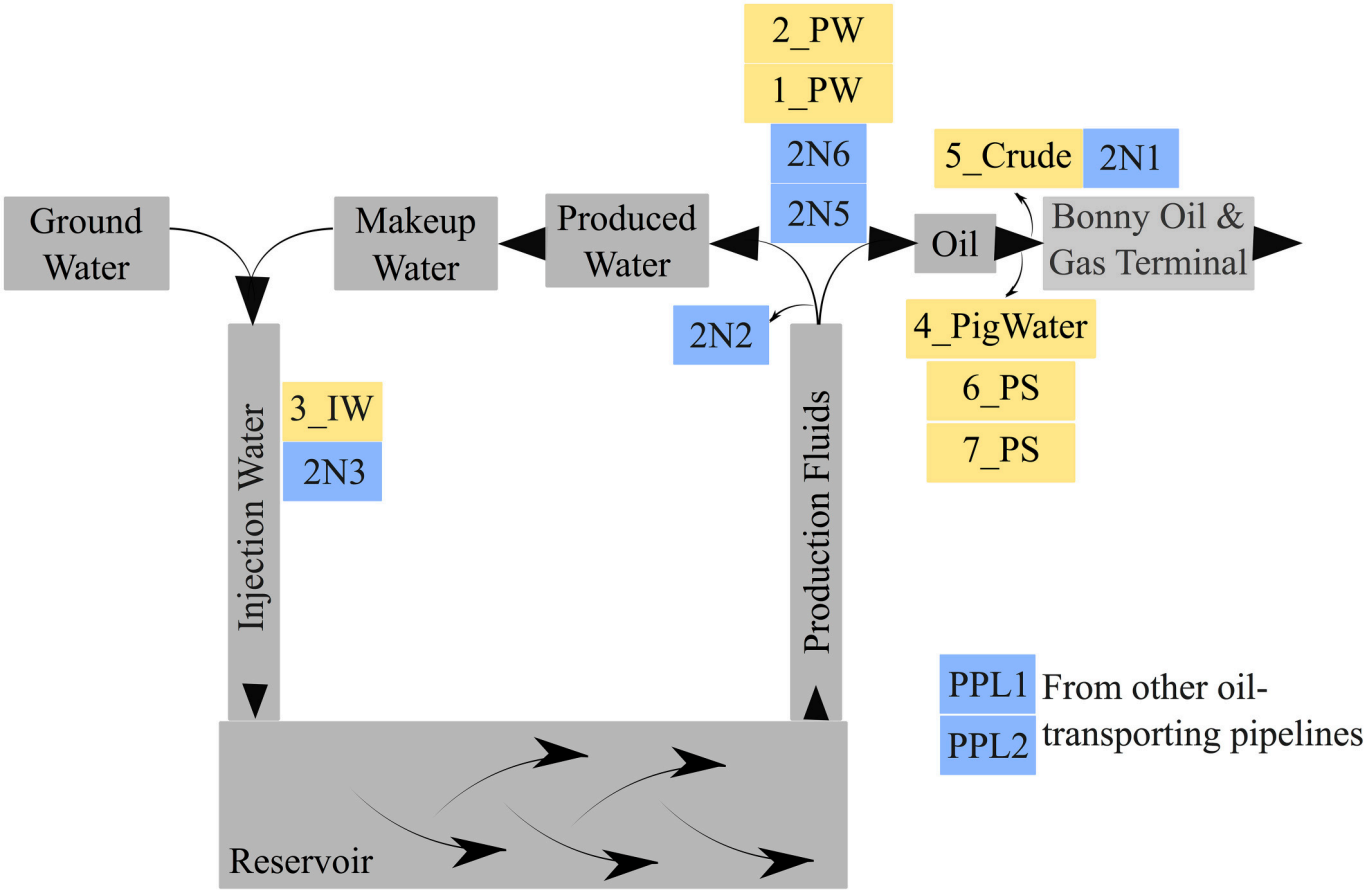
**Schematic diagram of the Obigbo oil field in Nigeria**. Samples received in 2013 are shown in yellow. 1_PW and 2_PW are produced waters, 3_IW is an injection water, 4_PigWater is water associated with a pigging operation, and 5_Crude is an oil and water mixture that is exported to an oil and gas terminal. 6_PS and 7_PS are pipeline pigging solids. Samples received from sampling in 2011 are indicated in blue. 2N2, 2N5, and 2N6 are production waters, 2N3 is an injection water and 2N1 is an oil and water mixture exported to an oil and gas terminal. PPL1 and PPL2 are pipeline pigging solids.

The production site that we have studied, the Obigbo oil field, is located onshore in the Niger delta region, near the city of Port Harcourt (Onwusiri et al., 2003). Sampling from the Obigbo field was done in 2013 and samples were shipped at ambient temperature to the University of Calgary, Calgary, AB, Canada for chemical and molecular analyses. Water samples were collected in 1 L bottles (Nalgene, Rochester, NY), filled to the brim and tightly capped to exclude oxygen ingress. In total, five water samples and two solids samples were collected. Sampling points are indicated in Figure 1. Of these samples, two were produced waters (PW) (1_PW, 2_PW), sampled from the surface facilities. A third sample was injection water (IW), the ground water sample injected into the reservoir for pressure maintenance (3_IW). A water sample associated with pigging operations, after the pig run, (4_PigWater) was also sampled, along with a sample of the water that is exported to the BOGT, 5_Crude. The pipeline solids (PS) samples were collected in plastic bags and were labeled 6_PS and 7_PS. Both of these were the solids resulting from a pigging operation and were in pipelines transporting product to the BOGT. Upon arrival at the laboratory, samples were stored in an anaerobic hood (Coy Laboratory Products, Grass Lake, MI) containing an atmosphere of 90% (vol/vol) N_2_, 10% (vol/vol) CO_2_ (N_2_-CO_2_), (Praxair, Calgary, AB) at room temperature.

### Chemical analyses

Chemical components in water, solids and oil samples may serve as indicators of corrosion or as a gauge for potential corrosion risk. Upon arrival at the University of Calgary, subsamples were taken and stored at −20°C, and were used for chemical analyses. Water samples were analyzed as sampled. Solids samples (5 g) were mixed with 10 mL MilliQ (Millipore, Etobicoke, ON) water, vortexed at high speed for 10 min and allowed to settle. The resulting supernatant was used for colorimetric assays; 1.5 mL of the supernatant was centrifuged at 13,300 rpm for 5 min; the supernatant was filtered through a 0.45 μm nylon filter (Millipore, Etobicoke, ON) and the filtrate was used for all liquid chromatography measurements.

Sulfide and ferrous iron concentrations were assessed using colorimetric assays (Trüper and Schlegel, 1964; Park et al., 2011). Ammonium concentrations were assayed using the indophenol method (American Public Health Association, 1992). The pH was measured using a pH meter (Orion VERSA STAR, Thermo Scientific, Beverly, MA). Conductivity was measured with a commercial probe and converted into molar equivalents of NaCl (Orion Conductivity Probe, Thermo Scientific, Beverly, MA).

Other chemical analyses were performed as previously described (Mand et al., 2014). To measure acetate concentrations, a Waters 515 model (Milford, MA) high-performance liquid chromatograph (HPLC) equipped with a Waters 2487 model UV detector set at 220 nm and an organic acid column (250 × 4.6 mm, Alltech Prevail) eluted with 25 mM KH_2_PO_4_ (pH of 2.5) were used. Samples (1 mL of each water sample, 1 mL of each filtrate for pigging solids samples) were centrifuged at 13,300 rpm for 5 min and 300 μL of the supernatant was acidified using 20 μL of 1 M H_3_PO_4_. 50 μL of this solution was injected and eluted at a flow rate of 1 mL/min. Sulfate concentrations were monitored using a Waters 600 model HPLC equipped with a Waters 432 conductivity detector and an IC-PAK anion column (150 × 4.6 mm, Waters) eluted with 24% (v/v) acetonitrile, 2% butanol and 2% borate-gluconate concentrate. Following centrifugation 100 μL of the sample supernatant was added to 400 μL of the acetonitrile solution; 50 μL was injected into the HPLC and eluted at 2 mL/min.

The presence of oil organics in pipeline solids samples was estimated by extraction with dichloromethane (DCM) and analysis using a gas chromatograph equipped with mass spectrometry (GC-MS) (7890A GC system, 5975C detector, Agilent, Santa Clara, CA) (Gieg et al., 2008; Agrawal et al., 2012). 10 mL of DCM was added to 1 g of pipeline solids sample 6_PS. This mixture was thoroughly mixed by vortexing at high speed to ensure that oil organics would become dissolved into the organic phase. Subsequently, the organic phase was dried over sodium sulfate and was concentrated to 1 mL under a headspace of N_2_. 1 μL of this was injected as previously described (Agrawal et al., 2012).

### Methanogenic incubations in synthetic oil field brine

Microbial methanogenesis was monitored in sterile 120 mL serum bottles. 50 mL of Coleville synthetic brine K (CSBK) medium (Callbeck et al., 2013), was dispensed into each bottle under a headspace of N_2_-CO_2_. CSBK medium contains (in g/L): NaCl (1.5), KH_2_PO_4_ (0.05), NH_4_Cl (0.32), CaCl_2_·2H_2_O (0.21), (MgCl_2_·5H2O (0.54) and KCl (0.1). After autoclaving under N_2_-CO_2_, a 30 mL solution of 1 M NaHCO_3_ was added, along with 1 mL of trace elements (Widdel and Bak, 1992), and 1 mL of a 1 M solution of Na_2_S. The pH was adjusted to be between 7.2 and 7.4. 2.5 mL of each Obigbo field water sample was added, amounting to a 5% inoculum. Two grams of each solids sample (as sampled) was added for the pigging sample incubations. Each incubation was done with and without carbon steel coupons (5 × 0.5 × 0.1 cm, American Society for Testing and Materials, ASTM, a366 containing 0.015% carbon) present, at 30°C with agitation at 100 rpm. Coupons were cleaned according to a National Association of Corrosion Engineers (NACE) standard protocol, and weighed thrice before incubation (NACE, 2013). Methane was measured as previously described, where 0.2 mL of the headspace was sampled periodically using a syringe that had been pre-flushed with N_2_-CO_2_ and injected into a gas chromatograph (Hewlett-Packard Model 5890) at 150°C, equipped with a flame-ionizing detector set to 200°C and a stainless steel column (0.049 × 5.49 cm, Porapak) (Mand et al., 2014). Following 8 weeks of incubation, coupons were again cleaned according to protocol, weighed thrice and the calculated weight loss was used to determine general corrosion rates (Park et al., 2011; NACE, 2013).

### Pipeline pigging solids incubation experiments

Pipeline solids samples were incubated anaerobically both in the presence and absence of carbon steel beads. The field sample was a powdery solid, with no aqueous phase when received. It was mixed with equal volumes of anaerobic, sterile MilliQ water before incubation. Close to 1 g of sample was mixed with 2.04 g of steel beads (Carbon steel precision ball, diameter 3/32 ± 0.001 inches, weight 0.055 g, Grainger, Richmond Hill, ON). These beads were sanded with 400 grit sandpaper and cleaned according to standard protocol and weighed thrice before incubation (NACE, 2013). Samples were incubated in 25 mL Hungate tubes, under an atmosphere of N_2_-CO_2_, at 30°C with agitation at 100 rpm. Methane was measured periodically by sampling 0.2 mL of the headspace using a syringe pre-flushed with anaerobic gas (N_2_-CO_2_) and injection into a GC-FID, as described above. Following incubation, the beads were cleaned according to standard protocol and corrosion rates were determined from metal weight loss. One replicate, representing each condition was used for DNA extraction and subsequent pyrotag sequencing, as described below.

### Microbial community analysis by pyrosequencing

Immediately upon arrival in the laboratory, 300 mL of each liquid field sample was filtered through a 0.2 μm membrane filter to collect biomass. These filters were frozen at −80°C for use in DNA extraction. At the same time, 1 g of each solids sample was also frozen at −80°C. Genomic DNA was isolated using a bead-beating procedure, as outlined by manufacturer instructions (FastDNA Spin Kit for Soil, MP Biomedicals, Santa Ana, CA). DNA was eluted in 75 μL of 10 mM Tris-Cl pH 8.5 buffer and quantified with the Invitogen Qubit fluorometer, using the Quant-iT dsDNA HS Assay Kit (Invitrogen, Burlington, ON). The V6-V8 regions of the 16S rRNA genes were amplified using a two-step PCR amplification using the TopTaq PCR Kit (Qiagen, Toronto, ON), as described earlier (Park et al., 2011). The first step (30 cycles) was done using 16S rRNA primers 926F (AAACTYAAAKGAATTGRCGG) and 1392R (ACGGGCGG TGTGTRC). These primers are suitable for the amplification of both bacterial and archaeal DNA. The second amplification step (10 cycles) used the first PCR product as a template and was done using FLX titanium amplicon primers 454_RA_X (primer 926F with a 25-nucleotide A-adaptor sequence: CGTATCGCCTCCCTCGCGCCATCAG and a 10 nucleotide multiplex identifier barcode sequence) and 454T_FwB (primer 1392R with a 25-nucleotide B-adaptor sequence: CTATGCGCCTT GCCAGCCCGCTCAG). The final 16S rRNA PCR amplicons were confirmed using agarose gel electrophoresis and subsequently purified using the QIAquick PCR Purification Kit (Qiagen, Toronto, ON). After measuring final concentrations of each PCR product using the Invitogen Qubit fluorometer again, amplicons were sent to Genome Quebec and McGill University Innovation Centre (Montreal, QC) for 16S rRNA pyrosequencing.

Analyses of data were done with the Phoenix2 software package, where data were subjected to stringent quality control (QC) checks (Soh et al., 2013). This included matching primer sequences, matching adaptor sequences, and removing chimeric sequences. Phoenix2 used a cutoff quality score for each sequence of 27 and a minimum length of each sequence of 200 base pairs (Kunin et al., 2010; Schloss et al., 2011). The remaining QC sequences were clustered into operational taxonomic units (OTUs) using average neighbor clustering at a distance of 5%. OTUs that contained less than 0.01% of total QC reads were filtered out. Each remaining OTU was subsequently assigned to a taxon by comparison with the non-redundant 16S rRNA small subunit SILVA database, release 108, using RDP classifier (SSU ref NR 108; http://www.arb-silva.de/no_cache/download/archive/release_108/Exports). A tree dendrogram was formed using the unweighted pair group method algorithm (UPGMA), where distances between communities was calculated using the Bray-Curtis coefficient in the Mothur software package (Schloss et al., 2009). The tree dendrogram was visualized using the MEGA4.2.2 program (Tamura et al., 2011).

### Culture-based microbial enumeration

The number of lactate-utilizing SRB and glucose-fermenting acid-producing bacteria (APB) present in all samples were assayed by inoculating commercial medium for SRB and APB growth (DALYNN, Calgary, AB) in a single inoculation series. One mL of each field water sample, or 1 mL of each pipeline solids sample mixture (0.5 mL sterile anaerobic MilliQ water and 0.5 g solids sample) was added to 9 mL of medium and mixed well. 1 mL from this tube was added to another tube containing 9 mL of medium until a dilution series up to 10^−10^ for each sample was achieved. The culturable population was estimated from the highest dilution showing growth after 30 days of incubation at 30°C. Growth for SRB was estimated from black precipitate formation in vials, due to iron sulfide formation. Growth of APB was estimated from a color change caused by the phenol red indicator in the medium, due to a change in pH caused by glucose fermentation to acidic products.

### Comparison with a sample from a Canadian oil field

Corrosion testing was also done using a produced water sample from a low-temperature (30°C) Canadian oil field. 120 mL serum bottles containing 50 mL of CSBK medium, as described above, and a headspace (70 mL) of N_2_-CO_2_ were used. These were inoculated with a produced water sample and an oil sample from the Medicine Hat Glauconitic C (MHGC) field, in Medicine Hat, AB, Canada (Voordouw et al., 2009). Serum bottles were amended and inoculated with: 1 mL oil only, 1 mL oil and 2.5 mL produced water (M_PW) or 2.5 mL M_PW only. Bottles containing the combination of oil and M_PW and bottles containing M_PW only were also incubated in the presence of carbon steel coupons (5 × 1 × 0.1 cm, ASTM a366 containing 0.015% carbon), prepared as described above (NACE, 2013). The headspace was sampled periodically to measure methane. Samples of the aqueous phase were also taken over time to measure acetate concentrations. Following the 6 week incubation at 30°C with agitation at 100 rpm, coupons were removed and cleaned and corrosion rates were determined using the resulting metal weight loss, as described above.

### Accession numbers

All samples were assigned a sample code for pyrotag sequencing, found in Tables S1, S2. The sequences from the raw reads are available from the Sequence Read Archive from the National Center for Biotechnological Information (NCBI), (under the following accession numbers): V30_1349 (SRR1508449), V30_1350 (SRR1508450), V30_1351 (SRR1508451), V30_1352 (SRR1508452), V30_1353 (SRR1508453), V30_1354 (SRR1508454), V39_1847 (SRR2189969), and V39_1850 (SRR2189970).

## Results

### Sample characteristics

All chemical analyses of the field samples are summarized in Table 1. The pH of all Obigbo samples was between 6.85 and 8.32. The molar equivalents of NaCl in these samples ranged from 1 to 220 mM. Sulfate levels were low in all water samples, but there was 8.9 and 11.6 mM found in the 6_PS and 7_PS pigging solids extracts, respectively. It is possible that this sulfate may be resulting from sulfide oxidation. A similar trend was seen for sulfide concentrations and ferrous iron concentrations. Almost no sulfide was present in the water samples, but 2.7–3.1 mM was found in the pigging solids extracts, as aqueous dissolved sulfide. The lack of sulfide in water samples may be due to oxidation during sampling or transport. Ferrous iron was not detected in water samples, but 160–690 μM was found in the pigging solids extracts. All samples contained very small amounts of ammonium (0–80 μM), acetate (0–1650 μM) and propionate (0–200 μM).

**Table 1:** **Chemical characteristics of the low temperature (40°C) Obigbo oil field water and pigging solids samples**.

**Sample**	**pH**	**NaCl (mM)**	**Sulfate (mM)**	**Sulfide (mM)**	**Ferrous Iron (μM)**	**Acetate (μM)**	**Propionate (μM)**
1_PW	8.13	174	0.09	0.02	0	0	0
2_PW	8.01	157	0	0.03	0	450	0
3_IW	7.66	1	0.036	0.02	0	0	0
4_PigWater	7.78	220	0	0.01	0	1650	0
5_Crude	8.32	199	0.005	0.02	0	0	0
6_PS	6.93	28	8.87	3.14	160	30	120
7_PS	6.85	32	11.57	2.71	690	40	200

### Microbial community composition

**Figure 2:**
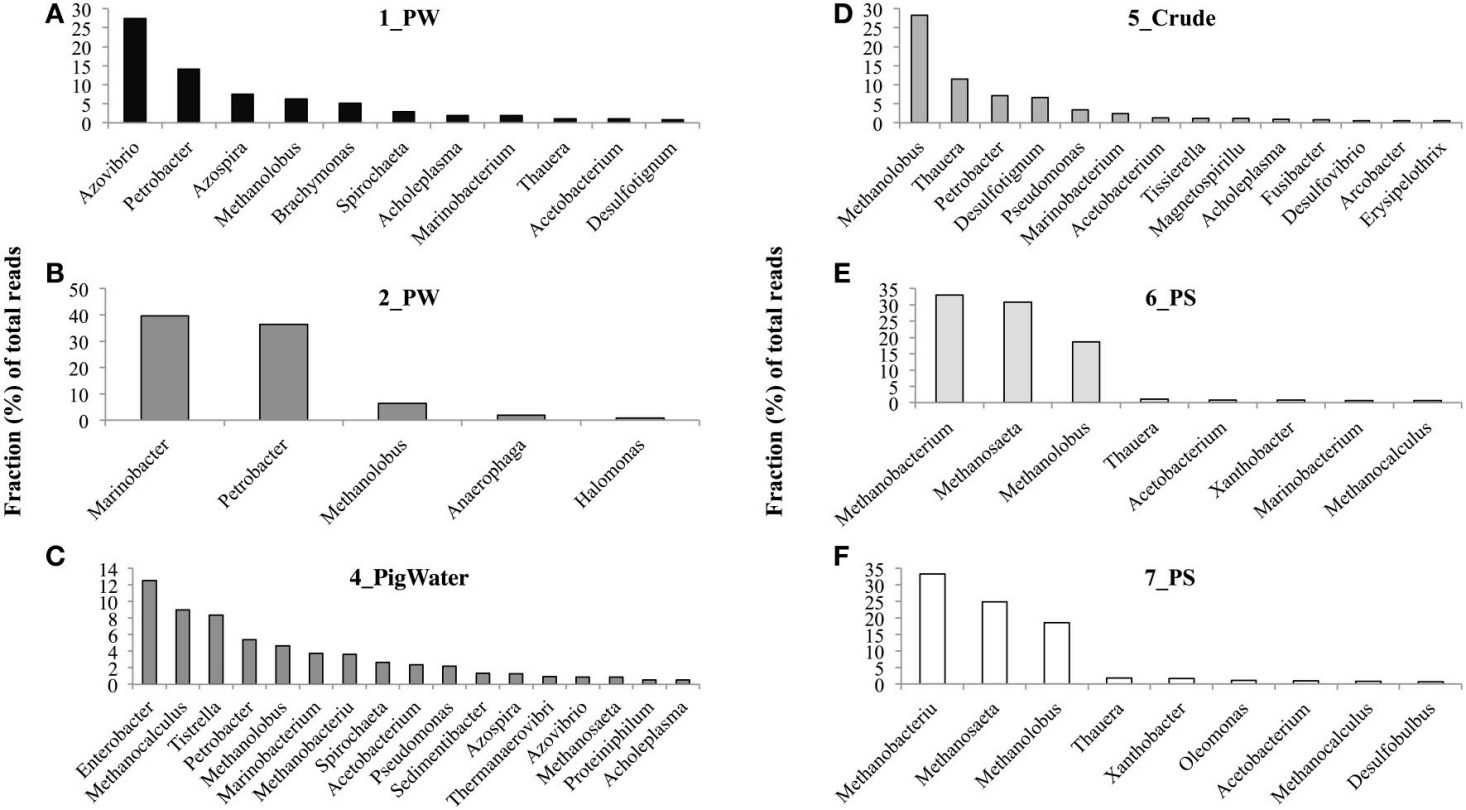
**Predominant genera identified in the Obigbo field samples, as determined by 454 pyrotag sequencing**. Only those with a representation of 0.5% or higher in produced water 1_PW **(A)**, produced water 2_PW **(B)**, pigging sample water 4_PigWater **(C)**, crude oil sample 5_Crude **(D)**, pipeline pigging sample 6_PS **(E)**, and pipeline pigging sample 7_PS **(F)** are shown.

A comprehensive microbial community composition was obtained using DNA sequencing technology. DNA was not successfully isolated from the 3_IW sample. This may have been caused by the low concentration of biomass in this water, as DNA isolation was also unsuccessful from this sample during 2011 sampling (Okoro et al., 2014). For the remaining field samples, a total of 6941 quality reads were obtained following 454-pyrotaq sequencing (Table S1). From this, it was seen that *Archaea* dominated the samples and the majority of these were methanogens (Figure 2). The pipeline pigging solids samples in particular harbored many methanogens. For example, the genus *Methanobacterium* formed between 33.0 and 33.3% of the total reads obtained for the two solids samples. *Methanobacterium* was also found in the pigging water sample, 4_PigWater, but in a smaller fraction (3.6%). Additionally, the genus *Methanosaeta* was abundant in the pigging solids samples, comprising 24.9–30.8% of all reads for these two samples. Other groups of methanogens such as the genus *Methanolobus* formed small portions of the population throughout all samples (4.6–18.7%). Noticeably, the genus *Methanocalculus* was more abundant in some water samples (4_PigWater: 9.0%) than in the pipeline solids samples (6_PS: 0.63%; 7_PS: 0.83%).

The produced waters from this field had other genera as the main community components (Figure 2). In the 1_PW sample, the genus *Azovibrio* formed 27.4% of the reads. The 2_PW sample was instead dominated by the genera *Marinobacter* (39.6%) and *Petrobacter* (36.3%), while *Thauera* were predominant in the 5_Crude sample (11.6%). All four of these genera are potential nitrate-reducing bacteria and are capable of hydrocarbon degradation. Small fractions of the field sample populations may belong to hydrocarbon degrading SRB as well. For instance, the genus *Desulfotigum* was found in 0.16–0.76% of the reads from the produced water and pigging solids samples. The genus *Acetobacterium* was also found in a small fraction (0.79–2.4%) of the reads for these samples. Cultured relatives of this organism are capable of metabolizing a variety of substrates and interestingly can convert CO_2_ and H_2_ into acetate using the acetyl-coenzyme A pathway (Schiel-Bengelsdorf and Dürre, 2012).

**Figure 3:**
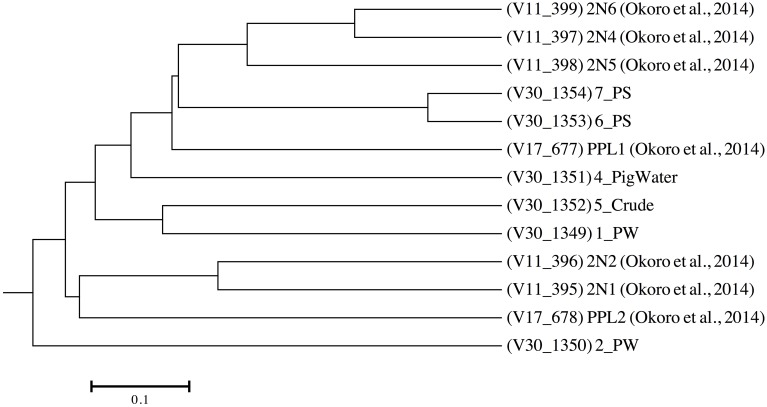
**Tree dendrogram showing the relationships between Obigbo field samples used in the present study (1_PW, 2_PW, 4_PigWater, 5_Crude, 6_PS, and 7_PS) and those used in a previous study (2N1, 2N2, 2N4, 2N5, 2N6, PPL1, and PPL2) (Okoro et al., 2014)**.

As mentioned, the Obigbo field has been previously sampled for microbial activity testing and microbial community composition analysis (Okoro et al., 2014). Sample sites and sample descriptions from this 2011 sampling trip can be found in Figure 1 and Table S3. The microbial communities present in the current field samples were compared with the previous study (Figure 3). The produced water samples from this study did not cluster with similar samples from the previous study, which is an indication that the microbial communities have changed over time at this site. Furthermore, the produced water samples (1_PW and 2_PW), which were taken from different parts of the production system, did not cluster together, possibly indicating that fluid processing during production leads to shifts in microbial populations. Also, the 4 solids samples, although taken from a different location each year, did not cluster together, demonstrating a change in the microbial community composition of pipeline biofilm associated microorganisms.

Culture-based incubations that target only certain culturable microorganisms are often correlated with corrosion in oil field systems. The numbers of lactate-oxidizing SRB and glucose-fermenting APB in each sample were estimated using a culturing assay, which is often used by pipeline operators in oil fields to assess microbial growth. APB were found in all Obigbo samples and ranged from 10^5^ to 10^7^ cells/mL (or g) of sample. SRB in the pipeline pigging solids samples (10^8^ cells/g) were higher than in the water samples (10^1^–10^5^ cells/mL). The low counts of APB (10^5^) and SRB (10^1^) in the 3_IW sample indicate low biomass concentrations, as was the case during previous studies (Okoro et al., 2014).

### Obigbo field sample incubations under methanogenic conditions

**Figure 4:**
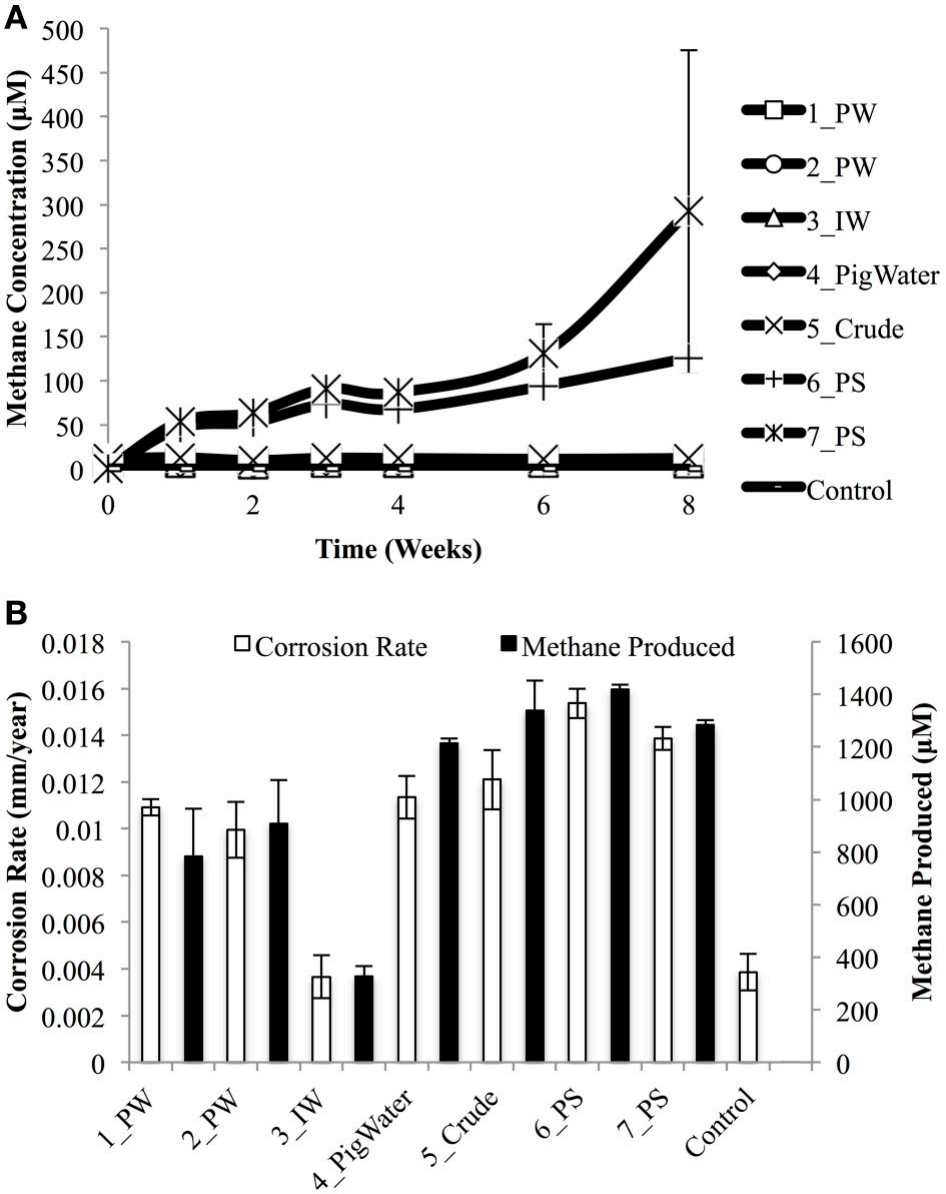
**Methane production over an 8 week incubation of Obigbo field samples in a synthetic oil field brine (CSBK) medium in the absence of carbon steel coupons (A)**. Maximum methane production and associated metal weight loss corrosion rates for the incubation of field samples in CSBK medium in the presence of carbon steel coupons **(B)**. Data represent results from two separate incubations containing two coupons each.

To test whether the different groups of hydrocarbon degrading organisms present in the field samples (Figure 2) were active and potentially involved in MIC, samples were incubated in a basal salts medium under an atmosphere of N_2_ and CO_2_, where in addition to the CO_2_ in the atmosphere, the hydrocarbons associated with the field samples served as carbon and energy sources. Incubation without the addition of carbon steel coupons yielded very low methane production (Figure 4A). Less than 12 μM methane was produced in each water sample enrichment. A maximum 126 ± 4.8 μM and 293 ± 183 μM methane was produced for pipeline solids samples 6_PS and 7_PS, respectively, but these values are very low compared to parallel incubations where steel coupons were present.

When the field samples were incubated with steel coupons, methane production could be correlated with corrosion rates (Figure 4B). A Pearson product-moment correlation coefficient was calculated to assess the relationship between methane production and corrosion rates. There was a positive correlation between the two variables (*r* = 0.95, *n* = 8, *p* = 0.71). The lowest methane concentration produced was in the incubation with the injection water sample (3_IW: 326 ± 55 μM) and this also showed the lowest corrosion rate (0.0037 ± 0.0013 mm/year). Conversely, the highest methane production was seen for incubations involving the pipeline pigging samples (6_PS: 1419 ± 26 μM and 7_PS: 1284 ± 24 μM). These incubations also showed the highest corrosion rates (6_PS: 0.0154 ± 0.0009 mm/yr and 7_PS: 0.0139 ± 0.0007 mm/yr). The control incubations containing medium only, with coupons showed no methane production and a very low corrosion rate (0.0039 ± 0.0011 mm/yr), similar to the injection water sample.

**Figure 5:**
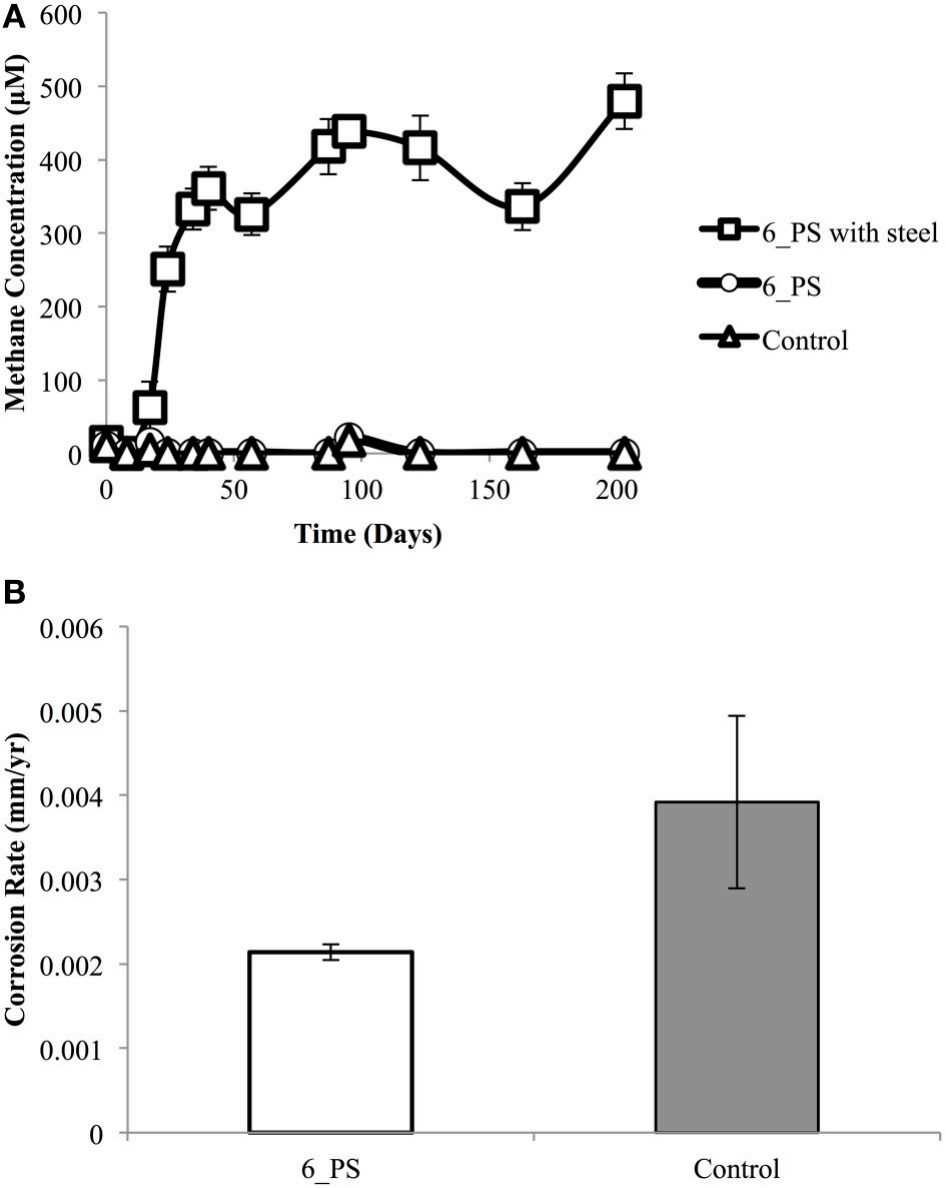
**Incubation of pigging solids sample 6_PS in the presence and absence of carbon steel beads**. Methane production was monitored over time **(A)**. Corrosion rates were determined following incubation **(B)**. Data are averages of three different incubations for each condition.

Further incubations were done using a pipeline pigging sample under methanogenic conditions. When the pigging solids sample, 6_PS, was incubated with carbon steel beads, up to 438 ± 18 μM methane was produced after 95 days of incubation (Figure 5A). In contrast, when the sample was incubated without any beads present, methane production was negligible. The corrosion rate obtained following this incubation was only 0.0021 ± 0.0001 mm/year, which is similar to the abiotic control (0.0039 ± 0.0010 mm/year) (Figure 5B). Acetate was not measured during these incubations, as very little aqueous phase was available for sampling.

Following the incubation period, 454-pyrotag sequencing was used to assess the community that emerged during this enrichment (Table S2). Of the reads obtained for the 6_PS incubation containing carbon steel beads, 55.98% were assigned to the order *Clostridiales*, which only formed 1.97% of the original 6_PS sample. This includes the genus *Acetobacterium*, which is known for the ability to make acetate from bicarbonate and steel (Kato et al., 2014; Mand et al., 2014). The fraction of the order *Methanosarcinales*, which contains the acetotrophic genus *Methanosaeta*, remained relatively high (19.75%) after incubation with steel, compared to the original field sample (50.75%).

### MHGC field sample incubations under methanogenic conditions

**Figure 6:**
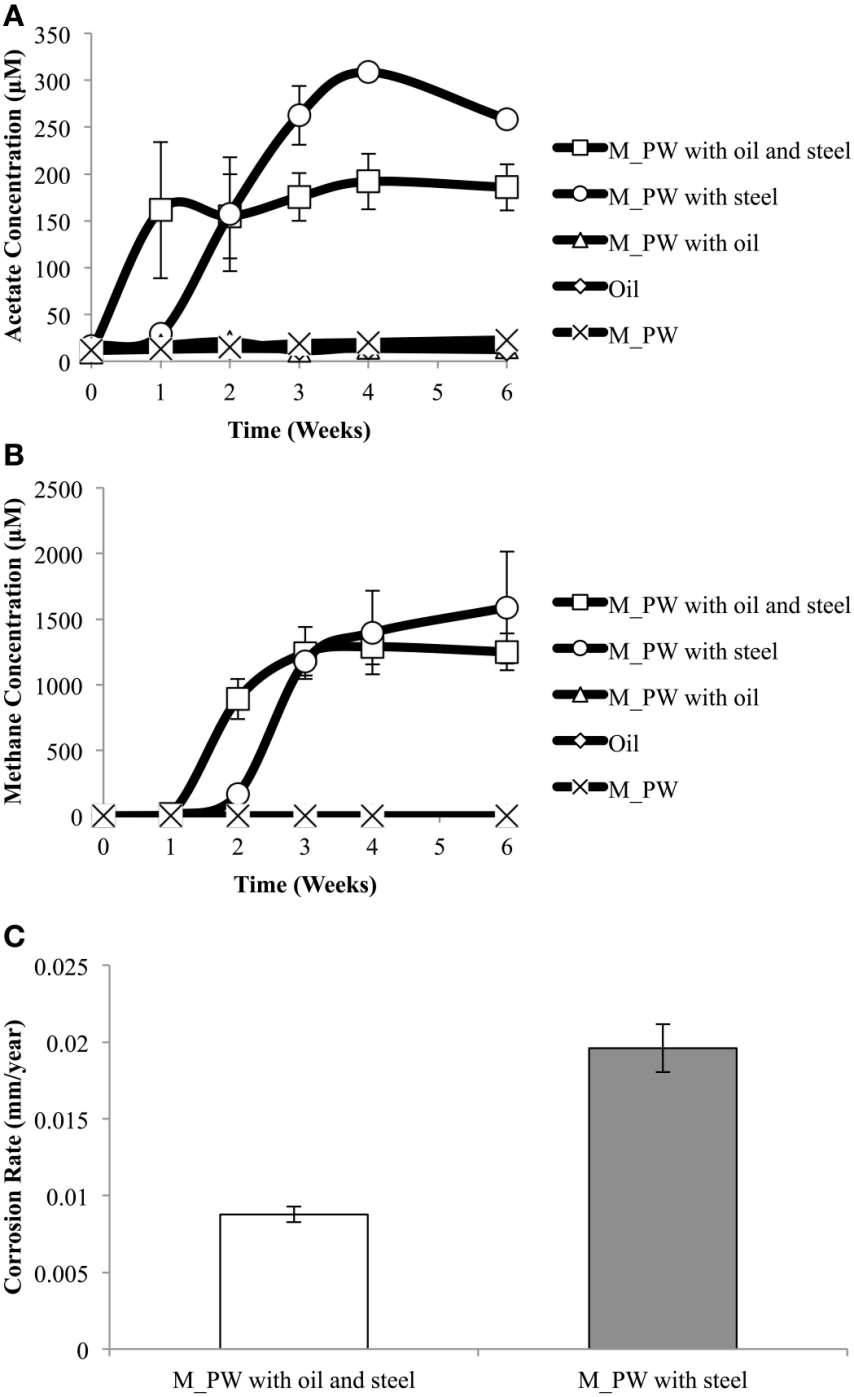
**A produced water sample from the MHGC field was incubated in a synthetic oil field brine (CSBK) medium with oil, with a metal coupon, with oil and a coupon, or by itself**. Acetate **(A)** and methane **(B)** production were measured over time. Corrosion rates **(C)**, as indicated were measured following incubation. Data represent the values from two different incubations for each condition.

To further understand the potential MIC scenarios seen with Obigbo incubations, samples from a different field were also used. A produced water sample from the MHGC field (M_PW) containing a methanogenic population (*Methanoculleus*, 70.2%) was used. This sample also contained the acetotrophic methanogen (*Methanosaeta*, 2.6%) and acetogenic bacteria (*Acetobacterium*, 0.13%). M_PW was incubated with and without oil, in the presence and in the absence of carbon steel coupons, using CSBK medium. When the M_PW sample (inoculum) was incubated by itself in medium, neither acetate nor methane was produced. These negative results were mirrored in the incubation of MHGC oil by itself in CSBK medium, without M_PW. Furthermore, when the M_PW and the MHGC oil were incubated together in CSBK medium, no microbial activity was witnessed. Acetate formation was only seen when a carbon steel coupon was added to the M_PW incubation (maximum production of 309 ± 5 μM) and when a carbon steel coupon was added to the produced water/oil incubation (maximum production of 192 ± 30 μM) (Figure 6A). Acetate production appeared to start earlier than methane production. Methane production was also only seen in incubations containing carbon steel. Methane production appeared to reach a plateau after 3 weeks of incubation, where a maximum of 1250 ± 141 μM was seen in the incubation of M_PW, oil and steel and a maximum of 1588 ± 427 μM was seen in the incubation of M_PW and steel (Figure 6B). Based on the concentrations of produced methane and acetate (Figures 6A,B) and assuming that iron was the reductant to make these from CO_2_, we calculate a theoretical corrosion rate of 0.028 mm/year in the absence of oil which is in good agreement with the experimental value (0.0196 ± 0.0016 mm/year) (Figure 6C). In the presence of oil we calculate a theoretical corrosion rate of 0.021 mm/year, which is higher than the measured 0.0088 ± 0.0005 mm/year (Figure 6C).

## Discussion

### Microbial communities and potential for biocorrosion

A previous study showed that the Obigbo oil field was dominated by methanogenic archaea, however it remained unclear how these may be thriving (Okoro et al., 2014). Our results confirm the presence of methanogens, specifically the genus *Methanobacterium*, an organism catalyzing methanogenesis using either hydrogen and bicarbonate or formate (4H_2_ + ${\text{HCO}}_{3}^{-}$ + H^+^ → CH_4_ + 3H_2_O or ${4\text{HCO}}_{2}^{-}$ + H_2_O + H^+^ → CH_4_ + ${3\text{HCO}}_{3}^{-}$) and also the genus *Methanosaeta*, which uses acetate to form methane (CH_3_COO^−^ + H^+^ → CO_2_ + CH_4_). There are several roles these microorganisms may play in MIC. Electrons from steel (iron) and protons from water form hydrogen on steel surfaces and the hydrogenotrophic methanogens use this hydrogen for their metabolism and thereby accelerate steel corrosion (Daniels et al., 1987). Other methanogens play a role in corrosion because of their ability to use electrons from iron directly to reduce bicarbonate (Dinh et al., 2004). Lastly, methanogens are able to use metabolic products produced by other, potentially corrosive organisms, such as the SRB (Zhang et al., 2003) or the acetogenic bacteria (Mand et al., 2014) seen in previous studies and thereby may contribute indirectly to MIC.

In incubations containing carbon steel coupons, methane production could be correlated to metal weight loss corrosion rates. There was a positive correlation between the maximum concentration of methane produced and corrosion rates measured for the 7 field samples and one abiotic control. Therefore, methane production was probably a result of using the iron, either through hydrogen production on the surface of the steel or as a direct electron donor to reduce bicarbonate and form methane.

Another potentially corrosive taxon in the microbial community of the Obigbo samples included the genus *Desulfotigum*, found in 0.16–0.76% of the population of the produced water and pigging solids samples. This toluene-degrading SRB was found in highest proportion in the sample associated with crude oil (5_Crude), where toluene may be abundant (Ommedal and Torsvik, 2007). In addition to producing sulfide, it can contribute to corrosion by degrading oil organics into potentially corrosive organic acids, similar to another report of SRB producing potentially corrosive organic acids (Lyles et al., 2014). There were many other bacterial genera capable of hydrocarbon degradation present in the samples as well. These include *Thauera* and *Petrobacter*, capable of producing organic acids as metabolic intermediates (Biegert et al., 1996; Salinas et al., 2004). Even modest concentrations of some organic acids, such as acetic acid, may be corrosive toward carbon steel (Liu et al., 2008), and in this way, these organisms may contribute to metal weight loss and therefore MIC at the Obigbo site. Furthermore, simple intermediates formed by these microorganisms could serve as metabolites for methanogenesis, in a process known as syntrophy (Gieg et al., 2014).

We were able to show that despite the presence of microorganisms known to catalyze syntrophic hydrocarbon degradation, the presence of methanogenic archaea and the availability of easy to degrade oil components in field samples, insignificant methane production was seen. This shows that methane production resulting from the syntrophic biodegradation of hydrocarbons was not occurring.

### Pipeline pigging solids samples

The two pipeline pigging solids samples (6_PS and 7_PS) were quite similar to one another. This was first noted in the sample chemistry. The solids samples contained the highest levels of sulfate potentially due to sulfide oxidation, which could lead to SRB growth, the highest concentrations of sulfide, a potential indicator of SRB activity and the highest concentrations of ferrous iron, a potential indicator of steel corrosion (Table 1).

The microbial communities of the two pipeline solids samples also harbored many of the same microorganisms (Figures 2E,F, 3). These were, in turn, quite different from the remaining water samples, including the pigging run water sample, 4_PigWater (Figure 2C). This may be an indication that the abundance and types of microorganisms found in the pipeline solids and potentially involved in corrosion are different from that in the planktonic population found in the pipeline fluid water samples.

When the community data were further examined, it was interesting to witness that between 70.8 and 93.5% of the microbial community of the water samples was classified as *Bacteria*, while the pigging samples were dominated by *Archaea* (79.5–85.6%). Therefore, the biofilm populations associated with pipeline surfaces, contained the highest proportion of methanogenic *Archaea* (Figure 2).

Microorganisms may predominantly live in biofilms on pipeline surfaces, instead of as planktonic free-flowing cells for a variety of reasons. A biofilm is a multifaceted structure, formed of different microbial cells embedded in a complex extracellular polymeric substance (EPS), and may be the preferred way for microbial growth in some natural environments (Costerton et al., 1995; Hall-Stoodley et al., 2004; Harrison et al., 2005). The cells within a biofilm are less vulnerable to eradication by chemical means or biopredation than free-swimming cells because they are protected by the EPS matrix and corrosion product matrix (Beech and Sunner, 2004). For example, when culture-based microbial enumerations were done, the highest counts of SRB and APB were found in the pigging solids samples, possibly because these cells were protected from chemical (e.g., biociding) and mechanical (e.g., pigging) processes. Living in this matrix is also beneficial for microbial cells because it serves to trap nutrients that may otherwise be lost in a fast-flowing pipeline. Evidence for this is suggested in the chemical data, which shows a higher concentration of ions, potential microbial nutrients, in the solids samples than the water samples (Table 1). In conclusion, it appears that pigging solids samples were more indicative than water samples for biocorrosion potential. In addition to taking aqueous samples when testing for microbes in the oil and gas industry, solids should also be sampled to obtain comprehensive microbial community data (Cote et al., 2014).

### Comparison with a Canadian low-temperature oil field

The MHGC field is a low temperature oil reservoir located near Medicine Hat, AB, Canada (Voordouw et al., 2009; Agrawal et al., 2012). The reservoir temperature is only 30°C, somewhat lower than the Obigbo field (40°C), but both are conducive to microbial growth. The MHGC field uses water flooding to produce oil, and the injection water contains a small amount (average 0.8 mM) of sulfate. Contrary to the Obigbo field, this field is flooded with nitrate to curb negative SRB activity (Voordouw et al., 2009; Agrawal et al., 2012). 454 pyrotag sequencing has shown that the majority of the microbial populations found in produced waters are methanogenic *Archaea* (Callbeck et al., 2013), similar to the Obigbo field.

Incubations of Obigbo pipeline pigging solids showed methane production in the presence of steel and a community composition enriched in hydrogenotrophic methanogens, acetogenic bacteria (*Acetobacterium*) and acetotrophic methanogens (*Methanosaeta*). These incubations were unable to uncover the role of acetate-producing organisms in MIC. When a produced water sample from the MHGC field was used to repeat the experiment, we were able to show methane and acetate production due to the presence of carbon steel coupons. Pyrotag sequencing of these samples revealed the same, potentially corrosive acetogenic bacteria (*Acetobacterium*) and acetotrophic methanogens (*Methanosaeta*). Since these same organisms were also found in the Obigbo pigging solids samples, these results suggested a role for these microorganisms in MIC at the Obigbo site.

While we have previously shown in pure culture studies that the acetate requirement of corrosive SRB may be fulfilled by corrosive acetogens (Mand et al., 2014), similar syntrophic roles may also be played in methanogenic consortia. In the present study it appears that in addition to corrosive hydrogenotrophic methanogens, corrosive acetogens may also produce acetate, which can then lead to acetotrophic methane production. The acetate may also serve as a carbon source for hydrogenotrophic methanogenesis.

## Conclusions

While many studies into biocorrosion continue to focus on microorganisms traditionally associated with MIC such as the SRB, we have shown here that oil field samples may contain an abundance of methanogenic archaea and hydrogenotrophic acetogens, which may also be active in steel corrosion. We were able to show that these groups were most active when incubated in the presence of carbon steel, indicating that the steel was preferred as the electron donor over oil organics by the methanogenic and acetogenic consortia present in these samples. Furthermore, we have also shown here, through genomic studies, that the microbial populations of pipeline solids samples may not be the same as the microbial population of liquid samples. In this study, it appears that the microbial communities most closely associated with pipeline surface biofilms were potentially more active in MIC than planktonic microorganisms associated with the water samples. This work emphasizes the need to obtain representative field samples in order to obtain accurate and useful MIC data.

## Author contributions

JM and HP designed and carried out the study, collected data, performed the analysis and wrote the manuscript. CO, BL, SS, LC, and GV contributed to data interpretation and preparation of the manuscript.

### Conflict of interest statement

The authors declare that the research was conducted in the absence of any commercial or financial relationships that could be construed as a potential conflict of interest.

## Footnotes

^1^Organization of Petroleun Exporting Countries (OPEC). Annual Statistical Bulletin (2015). Available at: www.opec.org.
