# Supplementary material for: Microbial Methane Production Associated with Carbon Steel Corrosion in a Nigerian Oil Field
Source: Front Microbiol. 2016 Jan 11;6:1538. doi: 10.3389/fmicb.2015.01538 (PMC4707241; doi:10.3389/fmicb.2015.01538)
Supplement: Supplementary file 1 [file Table1.DOCX]

**Supplementary Data**

**Table S1:** Phylogenetic classification of pyrotags for the Obigbo field samples. Taxa were assigned only to high quality reads and are ranked according to overall abundance across all samples. Only taxa appearing in greater than 0.1% of the total reads are shown here.

| *Sample Name* | 1_PW | 2_PW | 4_PigWater | 5_Crude | 6_PS | 7_PS | Total Number of Reads | Fraction (%) of Total Reads |
| --- | --- | --- | --- | --- | --- | --- | --- | --- |
| *Sample Code* | V30_1349 | V30_1350 | V30_1351 | V30_1352 | V30_1353 | V30_1354 |  |  |
| *Number of Quality Reads* | 1203 | 1374 | 1360 | 528 | 1269 | 1207 |  |  |
| **Taxon (Phylum_Class_Order_Family_Genus)** |  |  |  |  |  |  |  |  |
| Methanobacteria_Methanobacteriales_Methanobacteriaceae_Methanobacterium | 0.33 | 0.00 | 3.60 | 0.00 | 33.02 | 33.31 | 874 | 12.59 |
| Methanomicrobia_Methanosarcinales_Methanosarcinaceae_Methanolobus | 6.32 | 6.41 | 4.63 | 28.22 | 18.68 | 18.56 | 837 | 12.06 |
| Betaproteobacteria_Hydrogenophilales_Hydrogenophilaceae_Petrobacter | 14.05 | 36.32 | 5.37 | 7.20 | 0.00 | 0.17 | 781 | 11.25 |
| Methanomicrobia_Methanosarcinales_Methanosaetaceae_Methanosaeta | 0.00 | 0.00 | 0.88 | 0.00 | 30.81 | 24.86 | 703 | 10.13 |
| Gammaproteobacteria_Alteromonadales_Alteromonadaceae_Marinobacter | 0.00 | 39.59 | 0.00 | 0.00 | 0.16 | 0.25 | 549 | 7.91 |
| Betaproteobacteria_Rhodocyclales_Rhodocyclaceae_Azovibrio | 27.35 | 0.00 | 0.88 | 0.19 | 0.00 | 0.17 | 344 | 4.96 |
| Gammaproteobacteria_Enterobacteriales_Enterobacteriaceae_Enterobacter | 0.08 | 0.00 | 12.50 | 0.00 | 0.00 | 0.00 | 171 | 2.46 |
| *Kingdom* Bacteria | 2.91 | 0.29 | 8.82 | 0.19 | 0.24 | 0.25 | 166 | 2.39 |
| Methanomicrobia_Methanomicrobiales_Methanocalculus | 0.00 | 0.00 | 8.97 | 0.00 | 0.63 | 0.83 | 140 | 2.02 |
| Halobacteria_Halobacteriales_Deep_Sea_Hydrothermal_Vent_Gp_6(DHVEG-6) | 0.25 | 0.07 | 9.34 | 0.00 | 0.00 | 0.00 | 131 | 1.89 |
| Alphaproteobacteria_Rhodospirillales_Rhodospirillaceae_Tistrella | 0.00 | 0.07 | 8.38 | 0.19 | 0.00 | 0.08 | 117 | 1.69 |
| Betaproteobacteria_Rhodocyclales_Rhodocyclaceae_Thauera | 1.00 | 0.00 | 0.22 | 11.55 | 1.02 | 1.82 | 111 | 1.60 |
| Betaproteobacteria_Rhodocyclales_Rhodocyclaceae_Azospira | 7.56 | 0.00 | 1.25 | 0.00 | 0.00 | 0.00 | 108 | 1.56 |
| Gammaproteobacteria_Oceanospirillales_Oceanospirillaceae_Marinobacterium | 1.91 | 0.00 | 3.75 | 2.46 | 0.71 | 0.41 | 101 | 1.46 |
| Anaerolineae_Anaerolineales_Anaerolineaceae_uncultured | 0.17 | 0.00 | 0.66 | 0.19 | 3.70 | 3.15 | 97 | 1.40 |
| *Phylum* Bacteroidetes | 0.08 | 2.40 | 2.13 | 1.89 | 0.00 | 0.00 | 73 | 1.05 |
| Spirochaetes_Spirochaetales_Spirochaetaceae_Spirochaeta | 2.91 | 0.00 | 2.65 | 0.19 | 0.00 | 0.00 | 72 | 1.04 |
| Clostridia_Clostridiales_Family_XI_Incertae_Sedis | 1.66 | 0.07 | 2.94 | 0.76 | 0.32 | 0.25 | 72 | 1.04 |
| Clostridia_Clostridiales_Eubacteriaceae_Acetobacterium | 1.00 | 0.00 | 2.35 | 1.33 | 0.79 | 0.91 | 72 | 1.04 |
| Spirochaetes | 1.66 | 0.00 | 1.99 | 2.84 | 0.16 | 0.17 | 66 | 0.95 |
| Betaproteobacteria_Burkholderiales_Comamonadaceae_Brachymonas | 5.15 | 0.00 | 0.00 | 0.00 | 0.00 | 0.08 | 63 | 0.91 |
| *Phylum* Proteobacteria | 0.00 | 4.15 | 0.00 | 0.38 | 0.08 | 0.08 | 61 | 0.88 |
| Gammaproteobacteria_Pseudomonadales_Pseudomonadaceae_Pseudomonas | 0.33 | 0.00 | 2.21 | 3.41 | 0.24 | 0.33 | 59 | 0.85 |
| *Candidate Division* WS6 | 0.00 | 0.00 | 2.21 | 0.19 | 0.08 | 2.15 | 58 | 0.84 |
| Betaproteobacteria_Rhodocyclales_Rhodocyclaceae_uncultured | 4.66 | 0.00 | 0.07 | 0.00 | 0.00 | 0.00 | 57 | 0.82 |
| *Phylum* Bacteroidetes_VC2.1 | 1.16 | 0.00 | 0.07 | 6.63 | 0.00 | 0.00 | 50 | 0.72 |
| Deltaproteobacteria_Desulfobacterales_Desulfobacteraceae_Desulfotignum | 0.75 | 0.00 | 0.00 | 6.63 | 0.24 | 0.25 | 50 | 0.72 |
| Betaproteobacteria_Burkholderiales_Alcaligenaceae | 2.49 | 0.00 | 0.00 | 0.00 | 0.39 | 0.99 | 47 | 0.68 |
| Betaproteobacteria_Rhodocyclales_Rhodocyclaceae | 1.83 | 0.00 | 0.29 | 2.65 | 0.16 | 0.08 | 43 | 0.62 |
| Sphingobacteria_Sphingobacteriales_WCHB1-69 | 1.16 | 0.00 | 0.88 | 2.46 | 0.24 | 0.00 | 42 | 0.61 |
| Mollicutes_Acholeplasmatales_Acholeplasmataceae_Acholeplasma | 1.91 | 0.00 | 0.52 | 0.95 | 0.00 | 0.00 | 35 | 0.50 |
| Alphaproteobacteria_Rhizobiales_Hyphomicrobiaceae_Xanthobacter | 0.00 | 0.00 | 0.00 | 0.00 | 0.79 | 1.66 | 30 | 0.43 |
| Clostridia_Clostridiales_Lachnospiraceae_uncultured | 0.58 | 0.00 | 0.96 | 1.71 | 0.00 | 0.00 | 29 | 0.42 |
| Clostridia_Clostridiales_Family_XI_Incertae_Sedis_Sedimentibacter | 0.25 | 0.00 | 1.32 | 0.19 | 0.16 | 0.41 | 29 | 0.42 |
| Synergistia_Synergistales_Synergistaceae_Thermanaerovibrio | 0.50 | 0.07 | 0.96 | 0.00 | 0.16 | 0.41 | 27 | 0.39 |
| Bacteroidia_Bacteroidales_Marinilabiaceae_Anaerophaga | 0.00 | 1.82 | 0.00 | 0.00 | 0.00 | 0.00 | 25 | 0.36 |
| Deferribacteres_Deferribacterales_Deferribacteraceae | 0.00 | 1.60 | 0.00 | 0.38 | 0.00 | 0.08 | 25 | 0.36 |
| Betaproteobacteria | 0.42 | 0.44 | 0.00 | 1.71 | 0.08 | 0.17 | 23 | 0.33 |
| Methanomicrobia_Methanosarcinales | 0.00 | 0.00 | 0.00 | 0.00 | 1.10 | 0.75 | 23 | 0.33 |
| Gammaproteobacteria | 0.00 | 1.38 | 0.07 | 0.57 | 0.00 | 0.00 | 23 | 0.33 |
| Clostridia_Clostridiales | 0.17 | 0.00 | 0.81 | 0.38 | 0.24 | 0.33 | 22 | 0.32 |
| Betaproteobacteria_Hydrogenophilales_Hydrogenophilaceae | 0.50 | 0.80 | 0.00 | 0.57 | 0.00 | 0.00 | 20 | 0.29 |
| Bacteroidia_Bacteroidales_Rikenellaceae_vadinBC27 | 1.08 | 0.00 | 0.15 | 0.76 | 0.00 | 0.00 | 19 | 0.27 |
| Deltaproteobacteria_Desulfobacterales_Desulfobacteraceae | 0.00 | 1.24 | 0.00 | 0.00 | 0.00 | 0.08 | 18 | 0.26 |
| Deltaproteobacteria_Desulfobacterales_Desulfobulbaceae_Desulfobulbus | 0.00 | 0.00 | 0.37 | 0.38 | 0.24 | 0.66 | 18 | 0.26 |
| Alphaproteobacteria_Rhodospirillales_Oleomonas | 0.00 | 0.00 | 0.00 | 0.00 | 0.32 | 1.08 | 17 | 0.24 |
| *Phylum* Firmicutes | 0.58 | 0.00 | 0.44 | 0.38 | 0.00 | 0.08 | 16 | 0.23 |
| Bacteroidia_Bacteroidales_Porphyromonadaceae_Proteiniphilum | 0.17 | 0.22 | 0.52 | 0.19 | 0.08 | 0.00 | 14 | 0.20 |
| Lentisphaeria_Victivallales_Victivallaceae_uncultured | 1.08 | 0.00 | 0.00 | 0.00 | 0.00 | 0.00 | 13 | 0.19 |
| Alphaproteobacteria_Rhodobacterales_Rhodobacteraceae | 0.00 | 0.00 | 0.74 | 0.19 | 0.08 | 0.08 | 13 | 0.19 |
| Gammaproteobacteria_Oceanospirillales_Halomonadaceae_Halomonas | 0.00 | 0.80 | 0.15 | 0.00 | 0.00 | 0.00 | 13 | 0.19 |
| Actinobacteria_ Coriobacteriales_Coriobacterineae_Coriobacteriaceae | 0.00 | 0.00 | 0.15 | 0.00 | 0.47 | 0.33 | 12 | 0.17 |
| Lentisphaeria_WCHB1-25 | 0.33 | 0.00 | 0.52 | 0.00 | 0.00 | 0.00 | 11 | 0.16 |
| Sphingobacteria_Sphingobacteriales_SB-1 | 0.33 | 0.00 | 0.22 | 0.19 | 0.00 | 0.17 | 10 | 0.14 |
| Betaproteobacteria_Rhodocyclales_Rhodocyclaceae_Dechloromonas | 0.50 | 0.00 | 0.22 | 0.00 | 0.00 | 0.00 | 9 | 0.13 |
| Methanomicrobia_Methanomicrobiales_Methanolinea | 0.17 | 0.00 | 0.44 | 0.00 | 0.00 | 0.08 | 9 | 0.13 |
| Methanomicrobia_Methanomicrobiales_Methanomicrobiaceae_Methanoculleus | 0.17 | 0.00 | 0.29 | 0.00 | 0.24 | 0.00 | 9 | 0.13 |
| Thermoplasmata_WCHA1-57 | 0.17 | 0.00 | 0.44 | 0.00 | 0.08 | 0.00 | 9 | 0.13 |
| Methanomicrobia_Methanosarcinales_Methanosarcinaceae_Methanomethylovorans | 0.08 | 0.00 | 0.44 | 0.00 | 0.08 | 0.08 | 9 | 0.13 |
| Deferribacteres_Deferribacterales_Deferribacteraceae_Calditerrivibrio | 0.08 | 0.00 | 0.37 | 0.38 | 0.08 | 0.00 | 9 | 0.13 |
| *Candidate Division* OP9 | 0.00 | 0.00 | 0.07 | 0.00 | 0.47 | 0.17 | 9 | 0.13 |
| Clostridia_Clostridiales_Family_XII_Incertae_Sedis_Fusibacter | 0.08 | 0.00 | 0.15 | 0.76 | 0.00 | 0.08 | 8 | 0.12 |
| Gammaproteobacteria_Oceanospirillales_Halomonadaceae | 0.08 | 0.44 | 0.00 | 0.19 | 0.00 | 0.00 | 8 | 0.12 |
| Gammaproteobacteria_Pseudomonadales_Pseudomonadaceae | 0.08 | 0.00 | 0.00 | 1.33 | 0.00 | 0.00 | 8 | 0.12 |
| Methanobacteria_Methanobacteriales_Methanobacteriaceae_uncultured | 0.00 | 0.00 | 0.00 | 0.00 | 0.24 | 0.41 | 8 | 0.12 |
| Betaproteobacteria_Burkholderiales_Comamonadaceae | 0.58 | 0.00 | 0.00 | 0.00 | 0.00 | 0.00 | 7 | 0.10 |
| Spirochaetes_SHA-4 | 0.42 | 0.00 | 0.00 | 0.19 | 0.08 | 0.00 | 7 | 0.10 |
| Deltaproteobacteria_Desulfuromonadales_Desulfuromonadaceae_Desulfuromonas | 0.08 | 0.00 | 0.07 | 0.19 | 0.16 | 0.17 | 7 | 0.10 |
| Clostridia_Clostridiales_Family_XI_Incertae_Sedis_Tissierella | 0.00 | 0.00 | 0.07 | 1.14 | 0.00 | 0.00 | 7 | 0.10 |
| Clostridia_Clostridiales_Ruminococcaceae_Fastidiosipila | 0.00 | 0.00 | 0.00 | 0.38 | 0.16 | 0.25 | 7 | 0.10 |
