# Supplementary material for: Microbial Methane Production Associated with Carbon Steel Corrosion in a Nigerian Oil Field
Source: Front Microbiol. 2016 Jan 11;6:1538. doi: 10.3389/fmicb.2015.01538 (PMC4707241; doi:10.3389/fmicb.2015.01538)
Supplement: Supplementary file 2 [file Table2.DOCX]

**Supplementary Data**

**Table S2:** Phylogenetic classification of pyrotags for the 6_PS incubation. Taxa were assigned only to high quality reads and are ranked according to overall abundance across all samples. Only taxa appearing in greater than 0.1% of the total reads are shown here.

| *Sample Description* | Original 6_PS Sample | 6_PS Incubation With Steel | 6_PS Incubation Without Steel | Total Number of Reads | Fraction of Total Reads (%) |
| --- | --- | --- | --- | --- | --- |
| *Sample Code* | 1.795.V30_1353 | 2.853.V39_1847 | 2.853.V39_1850 |  |  |
| *Number of Quality Reads* | 1269 | 886 | 876 |  |  |
| **Taxon** |  |  |  |  |  |
| Methanomicrobia_Methanosarcinales_Methanosaetaceae_Methanosaeta | 30.65 | 19.75 | 3.31 | 593 | 19.56 |
| Clostridia_Clostridiales_Peptococcaceae_Desulfotomaculum | 0.00 | 41.76 | 18.84 | 535 | 17.65 |
| Methanobacteria_Methanobacteriales_Methanobacteriaceae_Methanobacterium | 33.10 | 0.00 | 3.54 | 451 | 14.88 |
| Methanomicrobia_Methanosarcinales_Methanosarcinaceae_Methanolobus | 18.60 | 0.00 | 0.00 | 236 | 7.79 |
| Alphaproteobacteria_Rhizobiales_Hyphomicrobiaceae_Xanthobacter | 0.79 | 0.00 | 23.63 | 217 | 7.16 |
| Deferribacteres_Deferribacterales_Deferribacteraceae | 0.08 | 8.24 | 4.00 | 109 | 3.60 |
| Clostridia_Clostridiales_Eubacteriaceae_Acetobacterium | 0.71 | 8.58 | 0.57 | 90 | 2.97 |
| Anaerolineae_Anaerolineales_Anaerolineaceae_uncultured | 3.70 | 0.56 | 4.11 | 88 | 2.90 |
| Thermotogae_Thermotogales_Thermotogaceae_Kosmotoga | 0.24 | 0.34 | 4.91 | 49 | 1.62 |
| Gammaproteobacteria_Pseudomonadales_Pseudomonadaceae_Pseudomonas | 0.24 | 0.90 | 2.40 | 32 | 1.06 |
| Synergistia_Synergistales_Synergistaceae | 0.00 | 2.60 | 0.80 | 30 | 0.99 |
| Clostridia_Clostridiales_Clostridiaceae_Clostridium | 0.00 | 2.48 | 0.80 | 29 | 0.96 |
| Thermotogae_Thermotogales_Thermotogaceae_Thermosipho | 0.00 | 1.69 | 1.03 | 24 | 0.79 |
| Epsilonproteobacteria_Campylobacterales_Campylobacteraceae_Arcobacter | 0.00 | 2.26 | 0.00 | 20 | 0.66 |
| *Candidate Division* OP9 | 0.47 | 0.00 | 1.60 | 20 | 0.66 |
| Methanobacteria_Methanobacteriales_Methanobacteriaceae_uncultured | 0.24 | 0.90 | 0.91 | 19 | 0.63 |
| Clostridia_Clostridiales_Family_XI_Incertae_Sedis_Sedimentibacter | 0.16 | 1.24 | 0.34 | 16 | 0.53 |
| Alphaproteobacteria_Rhodobacterales_Rhodobacteraceae_Roseobacter_clade | 0.08 | 0.23 | 1.48 | 16 | 0.53 |
| Methanomicrobia_Methanosarcinales | 1.26 | 0.00 | 0.00 | 16 | 0.53 |
| Actinobacteria_ Coriobacteriales_Coriobacterineae_Coriobacteriaceae_Eggerthella | 0.47 | 0.00 | 1.14 | 16 | 0.53 |
| Betaproteobacteria_Rhodocyclales_Rhodocyclaceae_Thauera | 1.02 | 0.00 | 0.34 | 16 | 0.53 |
| Deltaproteobacteria_Desulfuromonadales_Desulfuromonadaceae | 0.16 | 0.00 | 1.60 | 16 | 0.53 |
| Alphaproteobacteria_Rhodospirillales_Oleomonas | 0.32 | 0.00 | 1.26 | 15 | 0.49 |
| Sphingobacteria_Sphingobacteriales_WCHB1-69 | 0.24 | 0.00 | 1.14 | 13 | 0.43 |
| Spirochaetes_Spirochaetales_Spirochaetaceae_uncultured | 0.00 | 0.00 | 1.48 | 13 | 0.43 |
| *Phylum* Firmicutes | 0.00 | 0.34 | 1.03 | 12 | 0.40 |
| Synergistia_Synergistales_Synergistaceae_Aminobacterium | 0.00 | 0.79 | 0.46 | 11 | 0.36 |
| Clostridia_Clostridiales | 0.32 | 0.68 | 0.11 | 11 | 0.36 |
| *Kingdom* Bacteria | 0.32 | 0.56 | 0.23 | 11 | 0.36 |
| Clostridia_Clostridiales_Family_XI_Incertae_Sedis | 0.32 | 0.11 | 0.57 | 10 | 0.33 |
| Methanomicrobia_Methanomicrobiales_Methanocalculus | 0.63 | 0.00 | 0.23 | 10 | 0.33 |
| Deltaproteobacteria_Desulfovibrionales_Desulfovibrionaceae_Desulfovibrio | 0.08 | 0.34 | 0.57 | 9 | 0.30 |
| Gammaproteobacteria_1013-28-CG33 | 0.00 | 0.00 | 1.03 | 9 | 0.30 |
| Gammaproteobacteria_Oceanospirillales_Oceanospirillaceae_Marinobacterium | 0.71 | 0.00 | 0.00 | 9 | 0.30 |
| Clostridia_Clostridiales_Family_XI_Incertae_Sedis_Tissierella | 0.00 | 0.34 | 0.57 | 8 | 0.26 |
| Betaproteobacteria_Burkholderiales_Alcaligenaceae_Achromobacter | 0.39 | 0.00 | 0.34 | 8 | 0.26 |
| Deltaproteobacteria_Desulfobacterales_Desulfobulbaceae_Desulfobulbus | 0.24 | 0.00 | 0.57 | 8 | 0.26 |
| Gammaproteobacteria_Alteromonadales_Shewanellaceae_Shewanella | 0.00 | 0.00 | 0.91 | 8 | 0.26 |
| Actinobacteria_ Coriobacteriales_Coriobacterineae_Coriobacteriaceae | 0.00 | 0.23 | 0.57 | 7 | 0.23 |
| Clostridia_Clostridiales_Family_XII_Incertae_Sedis_Clostridiaceae_Acidaminobacter | 0.00 | 0.00 | 0.80 | 7 | 0.23 |
| Clostridia_Clostridiales_Peptococcaceae_Pelotomaculum | 0.00 | 0.00 | 0.80 | 7 | 0.23 |
| Clostridia_Thermoanerobacterales_Thermoanaerobacteraceae_Moorella | 0.00 | 0.68 | 0.00 | 6 | 0.20 |
| Synergistia_Synergistales_Synergistaceae_Thermanaerovibrio | 0.16 | 0.45 | 0.00 | 6 | 0.20 |
| Bacteroidia_Bacteroidales_Porphyromonadaceae | 0.16 | 0.11 | 0.34 | 6 | 0.20 |
| Thermoplasmata_WCHA1-57 | 0.08 | 0.00 | 0.57 | 6 | 0.20 |
| Caldisericia_Caldisericales_WCHB1-02 | 0.00 | 0.00 | 0.69 | 6 | 0.20 |
| Anaerolineae_Anaerolineales_Anaerolineaceae | 0.32 | 0.00 | 0.23 | 6 | 0.20 |
| Anaerolineae_Anaerolineales_Anaerolineaceae_Leptolinea | 0.00 | 0.00 | 0.69 | 6 | 0.20 |
| Clostridia_Clostridiales_Family_XI_Incertae_Sedis_Tepidimicrobium | 0.00 | 0.23 | 0.34 | 5 | 0.16 |
| Caldilineae_Caldilineales_Caldilineaceae_uncultured | 0.16 | 0.00 | 0.34 | 5 | 0.16 |
| Deltaproteobacteria_Desulfobacterales_Desulfobulbaceae | 0.00 | 0.00 | 0.57 | 5 | 0.16 |
| Deltaproteobacteria_Syntrophobacterales_Syntrophaceae_Desulfobacca | 0.00 | 0.00 | 0.57 | 5 | 0.16 |
| Gammaproteobacteria_Xanthomonadales_Xanthomonadaceae | 0.00 | 0.00 | 0.57 | 5 | 0.16 |
| Gammaproteobacteria_Enterobacteriales_Enterobacteriaceae_Escherichia | 0.00 | 0.45 | 0.00 | 4 | 0.13 |
| Sphingobacteria_Sphingobacteriales_vadinHA17 | 0.00 | 0.23 | 0.23 | 4 | 0.13 |
| Methanomicrobia_Methanomicrobiales_Methanomicrobiaceae_Methanofollis | 0.00 | 0.11 | 0.34 | 4 | 0.13 |
| Sphingobacteria_Sphingobacteriales | 0.00 | 0.11 | 0.34 | 4 | 0.13 |
| Alphaproteobacteria_Rhizobiales_Brucellaceae_Ochrobactrum | 0.16 | 0.11 | 0.11 | 4 | 0.13 |
| Actinobacteria_Actinobacteridae_Actinomycetales | 0.00 | 0.34 | 0.00 | 3 | 0.10 |
| Methanobacteria_Methanobacteriales_Methanobacteriaceae | 0.16 | 0.11 | 0.00 | 3 | 0.10 |
| Methanomicrobia_Methanomicrobiales_Methanomicrobiaceae_Methanoculleus | 0.16 | 0.11 | 0.00 | 3 | 0.10 |
| Clostridia_Clostridiales_Ruminococcaceae_Fastidiosipila | 0.08 | 0.11 | 0.11 | 3 | 0.10 |
| *Candidate Division* OP8 | 0.00 | 0.00 | 0.34 | 3 | 0.10 |
| Anaerolineae_Anaerolineales_Anaerolineaceae_Bellilinea | 0.00 | 0.00 | 0.34 | 3 | 0.10 |
| Anaerolineae_Anaerolineales_Anaerolineaceae_Longilinea | 0.00 | 0.00 | 0.34 | 3 | 0.10 |
| Betaproteobacteria_Hydrogenophilales_Hydrogenophilaceae_Petrobacter | 0.00 | 0.00 | 0.34 | 3 | 0.10 |
| Deltaproteobacteria_Desulfobacterales_Desulfobacteraceae_Desulfotignum | 0.24 | 0.00 | 0.00 | 3 | 0.10 |
| Deltaproteobacteria_Syntrophobacterales_Syntrophaceae_Syntrophus | 0.00 | 0.00 | 0.34 | 3 | 0.10 |
| Deltaproteobacteria_Syntrophobacterales_Syntrophobacteraceae_Desulforhabdus | 0.00 | 0.00 | 0.34 | 3 | 0.10 |
| Gammaproteobacteria_Chromatiales_Halothiobacillaceae_Halothiobacillus | 0.24 | 0.00 | 0.00 | 3 | 0.10 |
| Spirochaetes | 0.24 | 0.00 | 0.00 | 3 | 0.10 |
