# Supplementary material for: Microbial Methane Production Associated with Carbon Steel Corrosion in a Nigerian Oil Field
Source: Front Microbiol. 2016 Jan 11;6:1538. doi: 10.3389/fmicb.2015.01538 (PMC4707241; doi:10.3389/fmicb.2015.01538)
Supplement: Supplementary file 3 [file Table3.DOCX]

**Supplementary Data**

**Table S3:** Samples and sample descriptions from 2011 sampling study from Obigbo production facilities (as referred to in Figures 1 and 4).

| **Sample** | **Sample Description** |
| --- | --- |
| 2N3 | Groundwater (IW) |
| (V11_395) 2N1 | Produced water from delivery line |
| (V11_396) 2N2 | Produced water from HP separator |
| (V11_399) 2N6 | Produced water from inlet T1401 |
| (V11_398) 2N5 | Produced water from bulk discharge header |
| (V11_397) 2N4 | Produced water from sludge tank 1201B |
| (V17_677) PPL1 | Pipeline solids from water transporting pipeline |
| (V17_678) PPL2 | Pipeline solids from oil transporting pipeline |
